# All-optical control of exciton flow in a colloidal quantum well complex

**DOI:** 10.1038/s41377-020-0262-7

**Published:** 2020-02-27

**Authors:** Junhong Yu, Manoj Sharma, Ashma Sharma, Savas Delikanli, Hilmi Volkan Demir, Cuong Dang

**Affiliations:** 1 LUMINOUS! Centre of Excellence for Semiconductor Lighting and Displays, School of Electrical and Electronic Engineering, The Photonics Institute (TPI), Nanyang Technological University, 50 Nanyang Avenue, 639798 Singapore, Singapore; 2 Department of Electrical and Electronics Engineering and Department of Physics, UNAM-Institute of Materials Science and Nanotechnology, Bilkent University, Bilkent, 06800 Ankara Turkey; 3 School of Physical and Mathematical Sciences, Division of Physics and Applied Physics, Nanyang Technological University, 639798 Singapore, Singapore; 4 CINTRA UMI CNRS/NTU/THALES 3288, Research Techno Plaza, 50 Nanyang Drive, Border X Block, Level 6, 637553 Singapore, Singapore

**Keywords:** Lasers, LEDs and light sources, Optical physics

## Abstract

Excitonics, an alternative to romising for processing information since semiconductor electronics is rapidly approaching the end of Moore’s law. Currently, the development of excitonic devices, where exciton flow is controlled, is mainly focused on electric-field modulation or exciton polaritons in high-Q cavities. Here, we show an all-optical strategy to manipulate the exciton flow in a binary colloidal quantum well complex through mediation of the Förster resonance energy transfer (FRET) by stimulated emission. In the spontaneous emission regime, FRET naturally occurs between a donor and an acceptor. In contrast, upon stronger excitation, the ultrafast consumption of excitons by stimulated emission effectively engineers the excitonic flow from the donors to the acceptors. Specifically, the acceptors’ stimulated emission significantly accelerates the exciton flow, while the donors’ stimulated emission almost stops this process. On this basis, a FRET-coupled rate equation model is derived to understand the controllable exciton flow using the density of the excited donors and the unexcited acceptors. The results will provide an effective all-optical route for realizing excitonic devices under room temperature operation.

## Excitonics: all optical control

A convenient all-optical way to control exciton flow between two different colloidal quantum wells (CQWs) at room temperature has been demonstrated by scientists in Singapore. Junhong Yu and coworkers from Nanyang Technological University have shown how the combination of stimulated emission and Förster resonance energy transfer (FRET) can strongly manipulate the flow of excitons between closely spaced donor CdSe core-only CQWs and acceptor CdS/CdSe/Cds core-shell CQWs. A mixed solution of these CQWs in the molar ratio of 4:1 donor to acceptor was dried onto the surface of a hollow quartz capillary tube and excited by sub-nanosecond laser pulses. Experimental data at different excitation fluences clearly shows that stimulated emission can modulate the population of excited donors and unexcited acceptors which in turn affects the FRET process between the donors and acceptors, leading to exciton flow control.

## Introduction

Exciton-based solid-state devices have the potential to be essential building blocks^1,2^ for modern information technology to surpass the performance of conventional electronic devices since excitonics combines an ultrafast operation speed^3^ with a highly compact footprint^4^. Because of the direct interaction with photons, excitonic devices effectively eliminate the interconnection delay between photon-based information communication and electron-based information processing. Unlike photonic devices with a diffraction limit of the footprint, the exciton thermal de Broglie wavelength is extremely small (~10 nm) at room temperature. Exploiting excitonic devices requires the ability to control the excitonic properties (e.g., exciton flow, exciton recombination rates, or exciton energy) in an optically active medium^2,5^. Until now, a series of excitonic schemes have been used to achieve controllability based on exciton–photon polaritons^6,7^, coupled indirect excitons^4,5^, DNA nanotechnology^8,9^, or electric-field modulation^1,2^. Unfortunately, these configurations have restricted application in integrated circuits because they either sacrifice the operation speed due to the application of a gate voltage or are inherently complex due to the requirement of high-Q-factor microcavities, engineered electron/hole wavefunction overlap, or nanometer-scale precision DNA scaffolds.

Targeting the challenges described above, a scheme with an emphasis on all-optical control, bottom-up fabrication, and self-assembly offers a more attractive solution. Förster resonance energy transfer (FRET) is a particularly promising mechanism for excitonics, as dipolar coupling between a donor and an acceptor permits efficient and directed exciton flow in a simple solid mixture^10–13^. On the other hand, stimulated emission, in which the exciton recombination dynamics dramatically differs from that in spontaneous emission, is a possible control mechanism for exciton flow considering that the FRET process significantly depends on the density of excited donors and unexcited acceptors^14,15^. Colloidal quantum wells (CQWs) with high quantum efficiency, a tuneable energy bandgap, and solution processability have recently attracted broad research interest for optoelectronic devices^16–19^. Particularly, CQWs have exhibited robust excitons at room temperature with a binding energy up to 150 meV^16,18^, extraordinary optical gain performance^19,20^, and near-unity FRET efficiency^10,21^, making them promising as an ideal platform to achieve all-optical control of exciton flow using FRET.

Herein, we propose a strategy to control the exciton flow based on mediation of the FRET process by stimulated emission in a binary nanomaterial complex consisting of 4 monolayer (ML) core-only CdSe CQWs (donors) and 8 ML core-shell CdS/CdSe/CdS CQWs (acceptors). Specifically, at low pump fluence when the emission of both donors and acceptors is spontaneous (referred to as regime I), nearly 50% of the exciton population in the donors outflows into the acceptors via FRET. By increasing the pumping level to achieve stimulated emission in the acceptors (referred to as regime II), we can greatly enhance the exciton flow efficiency up to 90% since quick depletion of excitons in the acceptors significantly promotes the FRET process. Upon further increasing the fluence to initiate stimulated emission in the donors (referred to as regime III), the exciton flow towards the acceptors almost switches off because the stimulated emission rate in donors is much faster than the FRET rate. Furthermore, we develop a FRET-coupled kinetic model to identify the competing processes responsible for the variation in exciton flow. Our results, which demonstrate a prototype of an all-optical controllable exciton flow concept with multiple modulation stages, may inspire the design of all-optical excitonic circuits operating at room temperature.

## Results

**Fig. 1:**
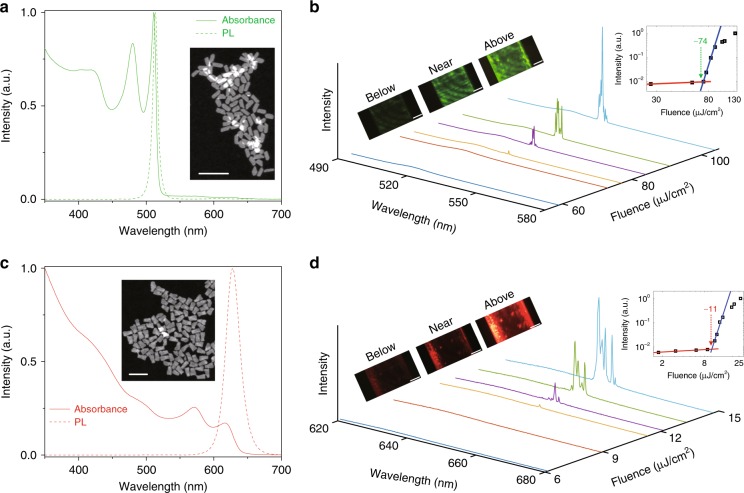
Optical characterizations of the pure donor and the pure acceptor. **a**, **b** Absorption (solid line) and emission spectra (dashed line) of 4 ML core-only CdSe CQWs, which serve as the donors (**a**) and 2/4/2 ML CdS/CdSe/CdS core-shell CQWs, which play the role of acceptors (**b**) dissolved in hexane. Inset: a TEM image of the CQWs, scale bar: 100 nm. **c**, **d** PL spectra of the donors (**c**) and the acceptors (**d**) coated inside a hollow capillary tube with an inner diameter of ~102 µm under different pump fluences (laser pulse: 355 nm, 500 ps). Left insets: optical images (with the pumping laser light filtered out) showing emission below, near, and above the threshold. Right inset: integrated intensity of the stimulated emission indicating a clear threshold behavior (integrating only the stimulated emission intensity)

The donor–acceptor pair used in this study is core-only CdSe and core-shell CdS/CdSe/CdS CQWs, composed of 4 MLs and 2/4/2 MLs in the vertical quantum-confinement direction, respectively (a detailed description of the synthesis is provided in the Methods). Two basic requirements for FRET to occur are spatial and energetic overlap^10,11,13,21^. The former implies that the distance between a donor and an acceptor should be less than the Förster radius, and the latter means that the spectral overlap between the donor’s emission and the acceptor’s absorption should be nonzero. The spatial overlap is easily satisfied in the CQW complex due to the extended plate geometry and large absorption cross-section (>10^−14^ cm^2^), as detailed previously^10,22^. In fact, the Förster radius for the CQW donor–acceptor pair^22^ has been measured to be as large as 10 nm. For the energetic overlap, as shown in Fig. 1a, c, the photoluminescence (PL) emission band of core-only CQWs fully matches the absorption features of core-shell CQWs; therefore, FRET can be expected in the binary complex.

Meanwhile, CQWs are also extraordinary optical gain materials, benefitting from (i) suppressed Auger recombination^16,17^ because momentum conservation is more difficult to satisfy than that in colloidal quantum dots and (ii) a giant modal gain coefficient^20,23,24^ since the increased exciton center-of-mass extension results in enhanced extinction coefficients. Before investigating the mediation of FRET by stimulated emission in the binary complex, the lasing action of each individual component is examined as a reference. We use a hollow quartz capillary tube to serve as a whispering-gallery-mode (WGM) resonator considering the ease of sample preparation (see Methods for details). The PL spectra of the CQWs filled in the tube with a diameter of ~102 µm are presented in Fig. 1b, d. For both core-only and core-shell CQWs, lasing is easily achieved, and the lasing peaks can be well assigned to resonance of the WGM cavity (see Fig. S1). It is worth noting that the core-shell CQWs exhibit a lower threshold than the core-only CQWs (11 versus 74 µJ/cm^2^, in the right inset of Fig. 1c, d). This can be easily understood because the CdS shell not only leads to the formation of a quasi-type II structure that further suppresses the Auger effect but also significantly increases the absorption cross-section and passivates the structure defects^24^, which is reflected in their quantum yield (QY) difference: ~45% and ~80% for the core-only and core-shell CQWs, respectively. Here, to extract the threshold, Voigt fitting is employed to separate the spontaneous and stimulated emission from the cavity spectra^25–29^.

**Fig. 2:**
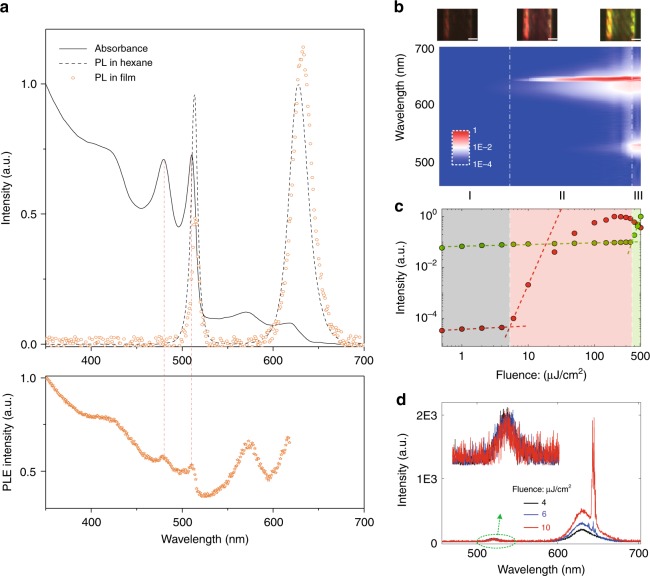
Optical characterizations of the mixed donor/acceptor. **a** The normalized absorption and PL spectra of the mixture in the solution and in the film, respectively (top panel). Bottom panel: PLE spectra of the mixed film with detection at the PL peak of core-shell CQWs (~628 nm). **b** The normalized contour map of emission spectra when the mixture is coated in the same hollow capillary tube described in Fig. 1. Emission intensity is plotted in log scale. White dashed lines indicate the thresholds of red lasing and green lasing. Top inset: optical images corresponding to spontaneous emission, red lasing, and dual lasing, respectively. **c** Integrated intensity of the stimulated emission as a function of the pump fluence for the donors (green dots/line) and the acceptors (red dots/line). Similar to Fig. 1, Voigt fitting is used to separate the spontaneous/stimulated emission from the cavity spectra and only the separated stimulated emission intensity is integrated. Three emission regimes are shaded in gray, light red, and light green, respectively. **d** Abnormal spontaneous emission behavior of the donor when pump fluence is around the threshold of red lasing. Inset: zoom in the donor’s spontaneous emission

The stimulated emission-mediated FRET process between the core-only and core-shell CQWs can now be investigated. We first present the PL and PL excitation (PLE) spectra in Fig. 2a to verify the exciton flow between the donors (core-only CQWs) and the acceptors (core-shell CQWs). Due to the large separation in the solution (i.e., low loading fraction of CQWs), FRET is negligible between the donors and the acceptors^10,12^. As the QYs and extinction coefficients of the two CQWs are different, the molar concentration ratio is intentionally adjusted (core-only:core-shell = 4:1) to ensure that the peak emission intensities from each component in the solution are relatively equal, which can be used as a reference to indicate the occurrence of exciton flow in the mixed film. The accelerated emission of donors and the delayed emission of acceptors observed in the time-resolved PL measurement, as shown in Fig. S2, is the first evidence for the FRET process in the mixed film. Using the equation^13^ $\eta ^{\mathrm{outflow}} = 1 - \tau _{\mathrm{donor}}^{\mathrm{mix}}/\tau _{\mathrm{donor}}^{\mathrm{pure}}$, where $\tau _{\mathrm{donor}}^{\mathrm{mix}}$ and $\tau _{\mathrm{donor}}^{\mathrm{pure}}$ are the lifetimes of donors in the mixed and pure films, respectively, we extract the efficiency at the designed ratio to be ~51.7% ($\tau _{\mathrm{donor}}^{\mathrm{mix}}$ and $\tau _{\mathrm{donor}}^{\mathrm{pure}}$ are ~0.63 and ~1.31 ns, respectively). By assuming that the emission intensity change of the donors is solely attributed to the FRET process, we can use this efficiency to normalize the emission intensity in the mixed film using^30,31^ $\eta ^{\mathrm{outflow}} = 1 - I_{\mathrm{donor}}^{{\mathrm{with}}\,{\mathrm{FRET}}}/I_{\mathrm{donor}}^{{\mathrm{without}}\,{\mathrm{FRET}}}$ (where *I* is the integrated emission intensity and details of the derivation are described in Supplementary Note 1). The results (orange dots in Fig. 2a) exhibit enhancement of acceptors’ emission and suppression of donors’ emission. Furthermore, the PLE results of the mixed film with detection at the PL peak of the core-shell CQWs (~628 nm) show two peaks corresponding to the absorption of the core-only CQWs. These three sets of experimental results (time-resolved PL, steady-state PL and PLE) unambiguously indicate that the excitons in the core-only CQWs outflow into the core-shell CQWs via the FRET process.

The mixed solution with the designed molar concentration ratio (donors:acceptors = 4 : 1) is loaded and then dried in the same hollow capillary tube corresponding to Fig. 1 and excited by the same sub-nanosecond laser pulses. The emission spectra from the WGM cavity (see Fig. 2b, the spectra for different pump fluences are shown in Fig. S3) exhibit three different regimes according to the increasing pump fluence: (I) at low pump fluence, only two spontaneous emission bands are presented; (II) as the pump fluence increases, red lasing from the acceptors is observed at a lower threshold of ~5.3 µJ/cm^2^ due to the indirect pumping from the donors via the FRET process (see Fig. 2c); and (III) the onset of green lasing is observed at very high pump fluence (~369 µJ/cm^2^, as in shown Fig. 2c), which is almost a five times increase from the threshold of the pure core-only CQWs since population inversion in the donors is largely hindered by the energy transfer. More significantly, the abnormal behavior of the spontaneous emission in the donors when the cavity emission crosses from regime I to regime II draws our attention. As shown in Fig. 2d, when the pump fluence is 4 µJ/cm^2^ (below the threshold of red lasing) and 10 µJ/cm^2^ (above the threshold of red lasing), we can barely observe an intensity increase of the spontaneous emission in the donors, which contradicts the common sense that in the spontaneous emission regime, the intensity linearly increases with the pump fluence^25–29^.

**Fig. 3:**
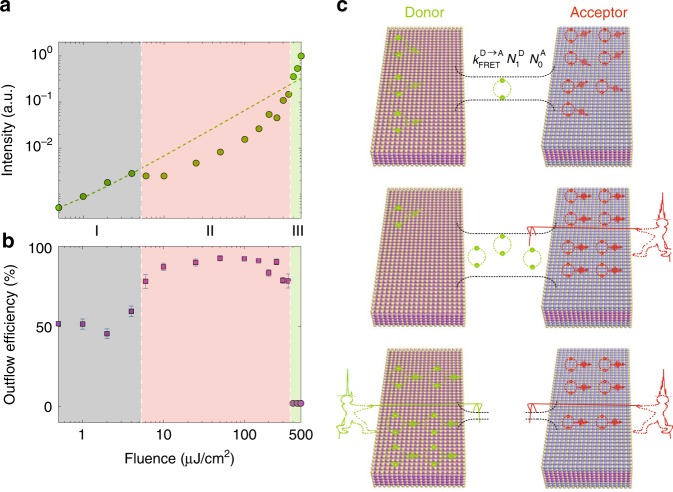
Controllable exciton flow between the donor–acceptor pair. **a** The normalized integrated intensity of donors’ spontaneous emission in the mixture coated in the hollow capillary tube. Green circle: the experimentally integrated intensity, which is are normalized by the maximum value; green dashed line: the linearly fitting to the integrated intensity in regime I. **b** The calculated exciton outflowing efficiency in the donor. Three emission regimes are shaded in gray, light red, and light green, respectively. In regimes I and II (squares), the outflowing efficiency is calculated based on the measured spontaneous emission intensity of donors. In regime III (circles), the outflowing efficiency is estimated from the lasing and FRET rate. **c** Illustration of controlling exciton flow by stimulated emission. $N_1^{\mathrm{D}}$ and $N_0^{\mathrm{A}}$ denote density of the excited donors and the unexcited (ground state) acceptors, respectively

To investigate the abnormal emission spectra of the binary complex in greater depth, we mainly focus on the donors’ spontaneous emission rather than the acceptors’ emission. The reason for this is that the change in donors’ spontaneous emission intensity can be directly used to characterize the exciton flow process, while the change in acceptors’ spontaneous emission is a convolution of the self-radiative recombination and the energy transfer process. In addition, in the majority of the applied pump fluence range, the donors’ PL is pure spontaneous emission, which ensures that exciton flow is the only process responsible for the intensity change. In Fig. 3a, we present the integrated intensity of donors’ emission (green circles), and a linear fitting to the first four integrated points is also presented as a reference (the green dashed line). We can observe that in regime I (the emission of both donors and acceptors is purely spontaneous, shaded in gray), the donors’ emission increases almost linearly with the pump fluence, suggesting that the FRET efficiency (i.e., the exciton outflow efficiency) is nearly constant, which matches the previous observations that the FRET process is independent of the low pump fluence in the spontaneous emission regime^13,32^. Once red lasing occurs (regime II, shaded in light pink), the increasing slope (i.e., *I*_donors_/fluence) of the spontaneous emission intensity of the donors dramatically drops, which indicates a significant enhancement of the exciton outflow efficiency. With a further increase in the pump fluence (regime III, shaded in light green), the integrated intensity of donors’ emission greatly increases due to the onset of lasing action.

We can now calculate the outflow efficiency of the donors’ excitons in regimes I and II, where the donors exhibit pure spontaneous emission, by using the same equation as discussed for Fig. 1: $\eta ^{\mathrm{outflow}} = 1 - I_{\mathrm{donor}}^{{\mathrm{with}}\,{\mathrm{FRET}}}/I_{\mathrm{donor}}^{{\mathrm{without}}\,{\mathrm{FRET}}}$. We still rely on the fact that the FRET process is the only reason for the spontaneous emission intensity change in donors. For absolute calibration of the FRET efficiency in the full range of pump fluences, we use the FRET efficiency of ~51.7% when the pump fluence is 0.5 μJ/cm^2^ as the reference point (measured in Fig. 2a and Fig. S2). As we can see in Fig. 3b, in the gray shaded regime (i.e., the acceptors’ emission is purely spontaneous), FRET naturally occurs between the donors and the acceptors with an efficiency of ~50%. Interestingly, with the onset of red lasing, the exciton population in the donors almost fully outflows into the acceptors with an efficiency above 90%. In regime III, when stimulated emission in donors occurs, we cannot use the same method to extract the exciton outflow efficiency since FRET is not the only process that changes the emission intensity. To address this issue, the ultrafast lasing dynamics in donors is resolved using a streak camera, and the lasing rate constant ($\gamma _{{\mathop{\rm{lasing}}\nolimits} }^{\mathrm{donors}}$) is evaluated as ~(16 ps)^−1^ (see Fig. S4 for details), which is more than 50 times greater than the FRET rate ($\gamma ^{\mathrm{FRET}}$) of ~(1.2 ns)^−1^ at our designed donor/acceptor ratio. By considering the consumption rate of excitons ($N_{\mathrm{donor}}^{\mathrm{mix}}$) in donors, ${\mathrm{d}}N_{\mathrm{donor}}^{\mathrm{mix}}/{\mathrm{d}}t = - \left( {\gamma _{\mathrm{donor}}^{\mathrm{pure}}\, + \,\gamma ^{\mathrm{FRET}}\, + \,\gamma _{{\mathop{\rm{lasing}}\nolimits} }^{\mathrm{donor}}} \right)N_{\mathrm{donor}}^{\mathrm{mix}}$, we can roughly estimate that the exciton outflow efficiency via FRET will be <2% due to the huge contrast between the FRET rate and lasing rate.

The efficiency variation in the binary CQW complex with respect to the occurrence of stimulated emission unambiguously implies modulation of the exciton flow in the donor–acceptor pairs. The qualitative explanation of the modulation mechanism is illustrated in Fig. 3c. The FRET-based exciton flow efficiency ($\eta ^{\mathrm{outflow}}$) depends on not only the FRET rate ($k_{\mathrm{FRET}}^{{\mathrm{D}} \to {\mathrm{A}}}$) but also the densities of the excited donors ($N_1^{\mathrm{D}}$) and the unexcited (ground state) acceptors ($N_0^{\mathrm{A}}$) as follows: $\eta ^{\mathrm{outflow}} \propto k_{\mathrm{FRET}}^{{\mathrm{D}} \to {\mathrm{A}}}N_1^{\mathrm{D}}N_0^{\mathrm{A}}$. When lasing occurs in the acceptors (regime II), the ultrafast consumption (~16 ps) of the excitons in the acceptors leads to an increase in $N_0^{\mathrm{A}}$ and therefore enhanced $\eta ^{\mathrm{outflow}}$. This is depicted in the middle panel of Fig. 3c, where the red boy (representing red lasing) is pulling very hard to accelerate the exciton flow. When lasing occurs in the donors (regime III), the ultrafast consumption of the excitons in the donors (quick suppression of $N_1^{\mathrm{D}}$ such that it approaches zero) leads to a negligible $\eta ^{\mathrm{outflow}}$. In other words, the FRET rate at the designed ratio in our CQW complex is not fast enough to compete with the stimulated emission in donors^33^, and as a result, the exciton flow is switched off in this regime.

**Fig. 4:**
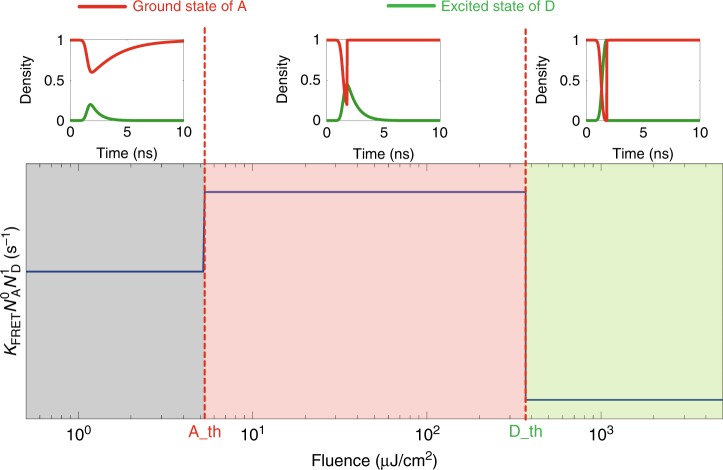
A kinetic model to explain the controllable exciton flow. The calculation results exhibit three different regimes, which agree with the experimental observations. Top inset: density of the donor’s excitons and unexcited (or ground stated) acceptor in three different regimes. A_th: the lasing threshold of the acceptor. D_th: the lasing threshold of the donor

Indeed, using a FRET-coupled kinetic equation model for the controllable energy transfer process mediated by stimulated emission^15,33^, we can reproduce the observed modulation of the exciton flow. For simplicity, we assume that the stimulated emission system in our CQWs is a three-level system. We model the stimulated emission process in the donors and the acceptors by introducing the ultrafast recombination rate *k*_lasing_ when the pump fluence is higher than the corresponding threshold. The full set of rate equations and related explanations are provided in Supplementary Note 2 in detail. To numerically evaluate our FRET-coupled model, we plug in the parameters extracted from the measurements (e.g., FRET rate and decay rates of the donor and the acceptor), while for the remaining parameters (i.e., the multiple-exciton state decay rate), we adopt the values from previous reports^14,33,34^. The numerical result shown in Fig. 4 illustrates the up and down trend of the exciton outflow efficiency presented in Fig. 3b. Specifically, stimulated emission can effectively modify the density of the excited donors and unexcited acceptors, leading to a considerable modulation of the exciton flow. It is worth mentioning that the exact exciton outflow efficiency predicted by this model differs from the experimental results due to our simplification. In particular, we have ignored the role of multiple-exciton states in the stimulated emission and the transition of the recombination rate from spontaneous emission to stimulated emission during the pump pulse width, which is considerably longer than many excitonic processes here. Nevertheless, our model can qualitatively reproduce the exciton flow trend from the donors to the acceptors demonstrated in our experiments.

## Discussion

The main advances of our work are discussed here. Inspired by the concept that the rate/efficiency of Förster-type exciton transfer is significantly dependent on the population overlap between excited donors and unexcited acceptors, we have demonstrated room temperature manipulation of exciton flow in a binary colloidal nanomaterial complex via an all-optical route. Specifically, ultrafast modulation of the density of excited donors and unexcited acceptors by stimulated emission plays a key role in controlling the exciton flow. Three distinct exciton flow regimes with efficiencies of ~50%, ~90%, and ~2% are triggered by the continuous transition from spontaneous emission to the acceptors’ stimulated emissions and then to dual stimulated emission (both the donors and the acceptors). This active excitonic control in an all-optical device scheme (i.e., in the WGM laser configuration) not only offers a platform to gain deeper insight into the FRET physics but also is highly preferable for excitonic-based information processing to achieve all-optical excitonic circuits.

## Materials and methods

### Material synthesis

Four ML core-only CdSe CQWs are synthesized according to our previously published method^20^. Eight ML core-shell CdS/CdSe/CdS CQWs are also synthesized according to the previously published literature^17^. All synthesized samples are cleaned with ethanol to remove excess ligands and finally dispersed in hexane for further experiments and measurements.

### Optical characterization of CQWs

The absorption spectra of CQWs in hexane are measured by a ultraviolet-visible spectrophotometer (Shimadzu, UV-1800). The PL of CQWs and PLE spectra of CQWs are recorded using a spectrofluorophotometer (Shimadzu, RF-5301PC, excitation wavelength for PL: 355 nm). The QY of CQWs in hexane is measured with an integrating sphere and calculated as the ratio of the absolute photon number of emission to that of absorption. The accuracy of the QY measurement is verified using rhodamine 6G, whose QY of 94.3% measured in our setup is found to be in good agreement with the standard value of 95%.

### Preparation of the donor–acceptor mixture

Core-only and core-shell CQWs dissolved in hexane are prepared at the same molar concentration (10^−4^ mol/L). Solutions with different donor:acceptor molar ratios are mixed using ultrasonication for 5 min. Specifically, for donor:acceptor = 4:1, the two emission bands from the mixed solution exhibit almost equal intensities.

### Loading of the mixture into the capillary tube

Coating optical gain materials (CQWs) onto a microcavity (quartz capillary tube) is adopted in this study. The donor–acceptor mixture is infiltrated into the tube by the capillary effect. To ensure that the solution forms a uniform film inside the tube, the tube is placed on a horizontal plate until the solvent is evaporated. After that, the two ends of the tube are sealed with wax to avoid contamination.

### PL dynamics measurement

Time-resolved PL spectroscopy is performed with a Becker & Hickl DCS 120 confocal scanning FLIM system with a laser pulse (70 ps) at a wavelength of 375 nm with a repetition rate of 20 MHz. For all the time-resolved PL measurements, the collection time is 180 s. For lifetime measurements, the mixture is spin coated (at 1500 r.p.m. for 2 min) onto a precleaned quartz substrate to ensure optically clear (minimum scattering) and uniform solid films.

## Supplementary information

SUPPLEMENTARY INFORMATION for All optical control of exciton flow in a colloidal quantum well complex
