# Supplementary material for: All-optical control of exciton flow in a colloidal quantum well complex
Source: Light Sci Appl. 2020 Feb 27;9:27. doi: 10.1038/s41377-020-0262-7 (PMC7046609; doi:10.1038/s41377-020-0262-7)
Supplement: Supplementary file 1 — SUPPLEMENTARY INFORMATION for All optical control of exciton flow in a colloidal quantum well complex [file 41377_2020_262_MOESM1_ESM.docx]

**SUPPLEMENTARY INFORMATION FOR**

All optical control of exciton flow in a colloidal quantum well complex

*Junhong Yu^1^, Manoj Sharma^1,2^, Ashma Sharma^1^, Savas Delikanli^1,2^, Hilmi Volkan Demir^*1,2,3^, Cuong Dang**^*1,4^*

^1^LUMINOUS! Centre of Excellence for Semiconductor Lighting and Displays, School of Electrical and Electronic Engineering, The Photonics Institute (TPI), Nanyang Technological University, 50 Nanyang Avenue, 639798, Singapore

^2^Department of Electrical and Electronics Engineering and Department of Physics, UNAM-Institute of Materials Science and Nanotechnology, Bilkent University, Bilkent, Ankara, 06800, Turkey

^3^School of Physical and Mathematical Sciences, Division of Physics and Applied Physics, Nanyang Technological University, 639798, Singapore

^4^CINTRA UMI CNRS/NTU/THALES 3288, Research Techno Plaza, 50 Nanyang Drive, Border X Block, Level 6, 637553, Singapore

* Email: [volkan@stanfordalumni.org](mailto:volkan@stanfordalumni.org); [hcdang@ntu.edu.sg](mailto:hcdang@ntu.edu.sg)

# Supplementary Figure 1


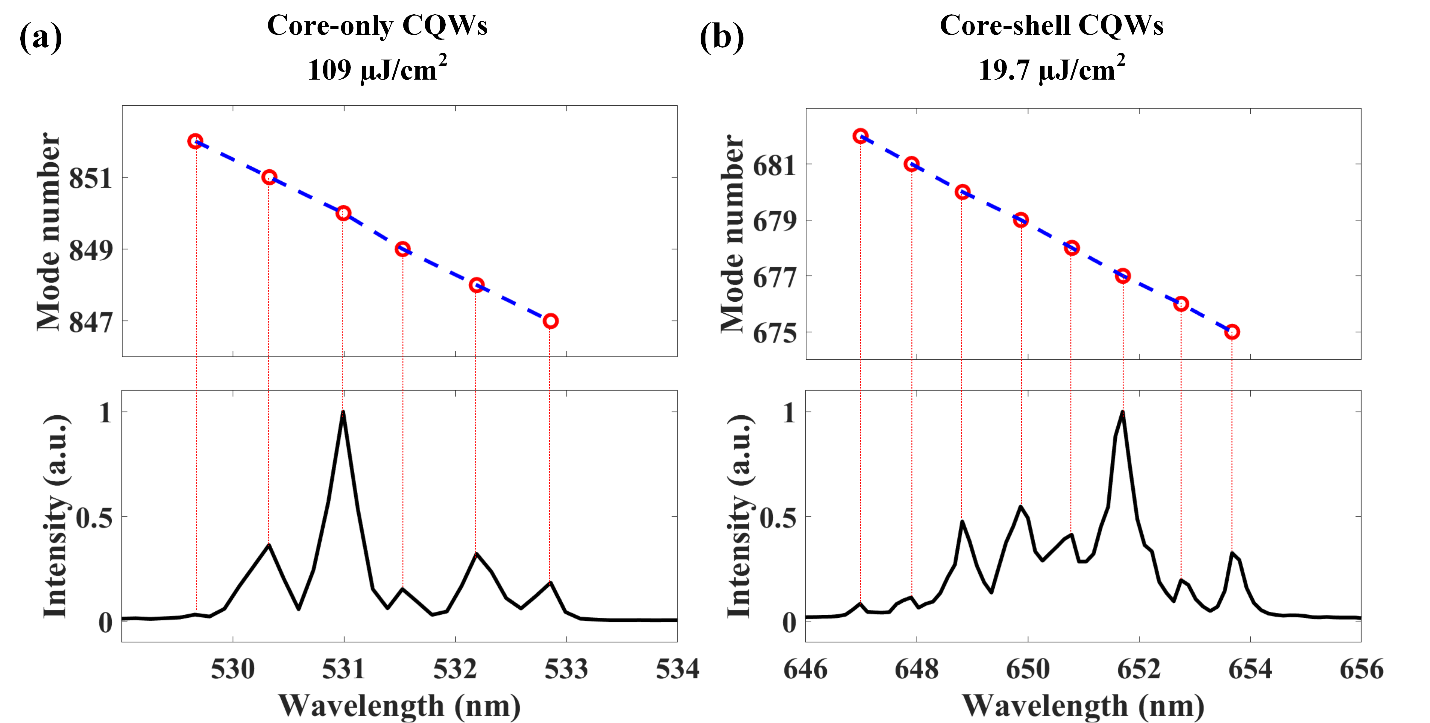


**Figure S1.** Lasing modes of both core-only **(a)** and core-shell **(b)** CQWs are well assigned according to the first radial order WGM model (*r* = 1), supporting WGM lasing mechanism. The resonant condition is given by: $m\lambda_{m}=\pi Dn_{eff}$. Where *m* is the mode number, *D* is the inner diameter of our tube (~ 102±2 µm), *n_eff_* is the effective refractive index of optical gain material.

# Supplementary Figure 2


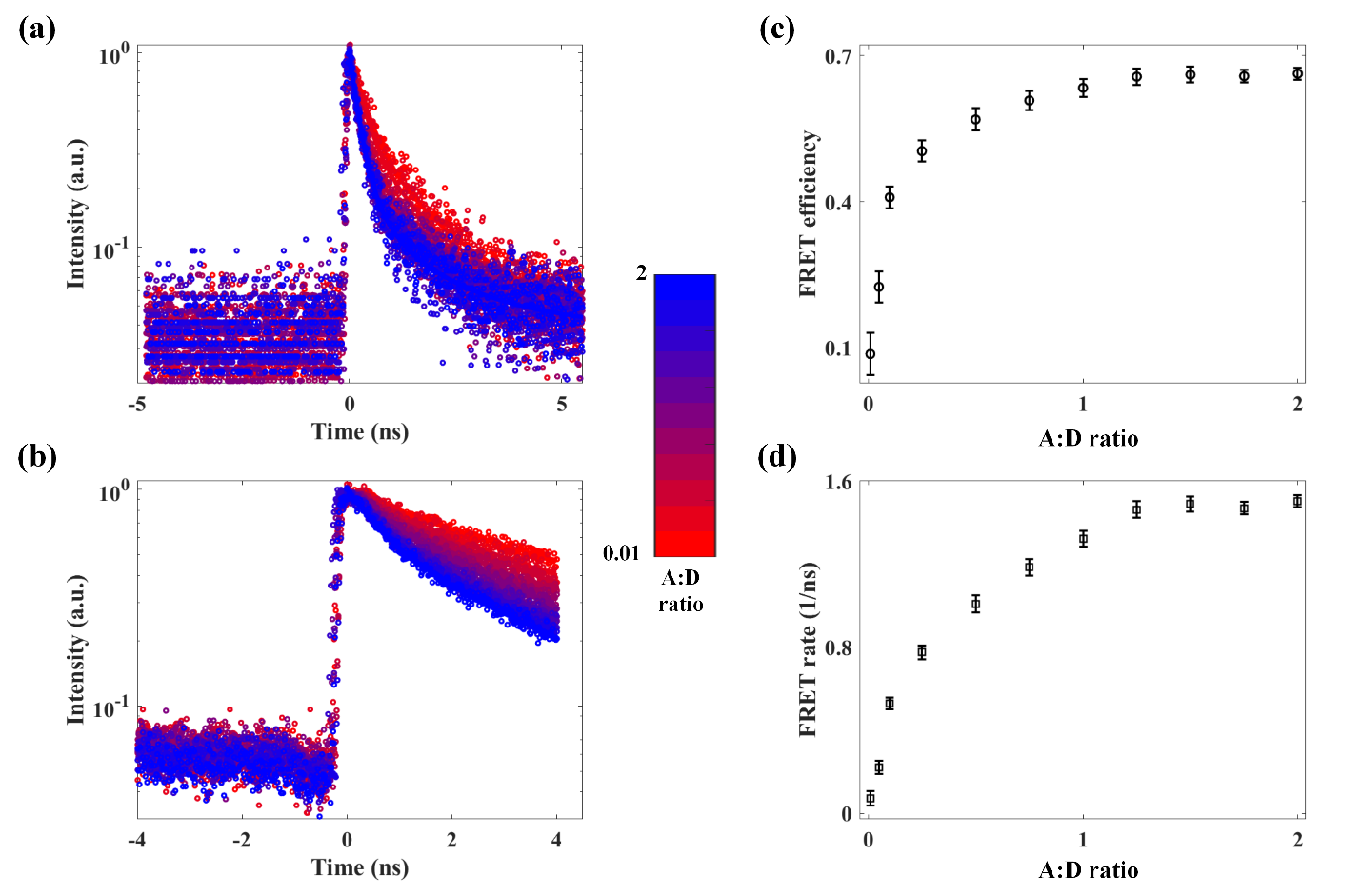


**Figure S2. Time-resolved PL measurement.** In the exciton flow control experiment, we need to choose a moderate A:D molar ratio (i.e., 0.1~0.4, 0.25 is adopted in the main text) to ensure that all three regimes will be observed without thermal degradation. A higher A:D molar ratio will enhance the exciton flow efficiency in regime I and regime II, while have negligible effect on the exciton flow efficiency in regime III. **(a)** The PL decay of the donor at different mixture ratio. When the acceptor ratio increases, the exciton lifetime of the donor becomes faster. **(b)** The PL decay of the acceptor at different mixture ratio. When the acceptor ratio decreases, their exciton lifetime becomes significantly extended. **(c)** The calculated FRET efficiency based on the reduced donor’s lifetime^1^. For our designed ratio (0.25), the calculated efficiency of ~ 51.7% is used to normalize the emission intensity in the mixed film, as discussed in Fig. 2. **(d)** The calculated FRET rate based on the reduced donor’s exciton lifetime^2^.

# Supplementary Figure 3


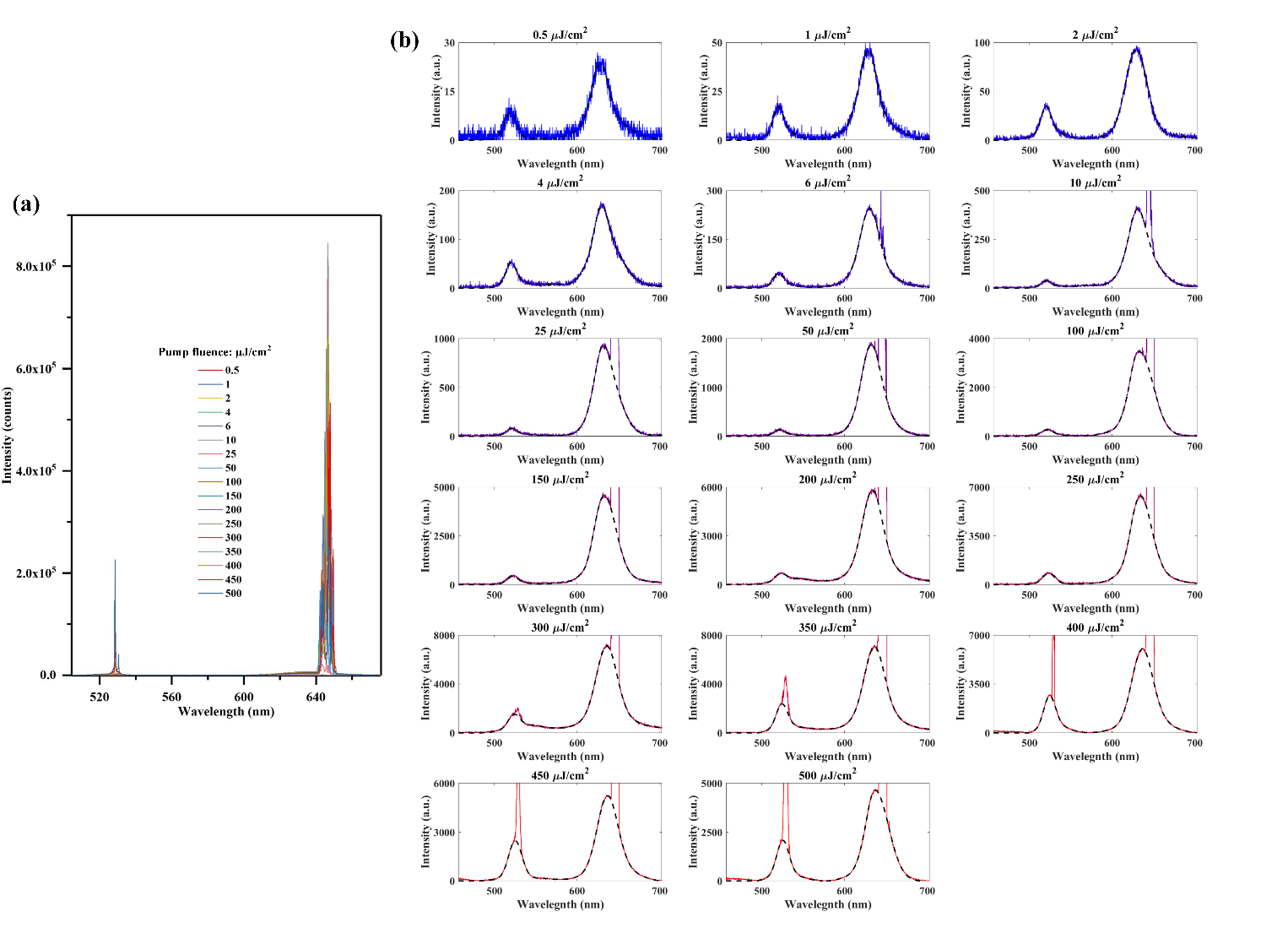


**Figure S3.** Emission spectra of the binary CQW complex in the WGM cavity with different pump fluences. (a) The emission spectra plot together. (b) The individual spectra under different pump fluence, the maximum intensity is limited to exhibit spontaneous emission. Voigt fitting (the black dashed line) is adopted to extract the lasing threshold for donors and acceptors^2^.

# Supplementary Figure 4


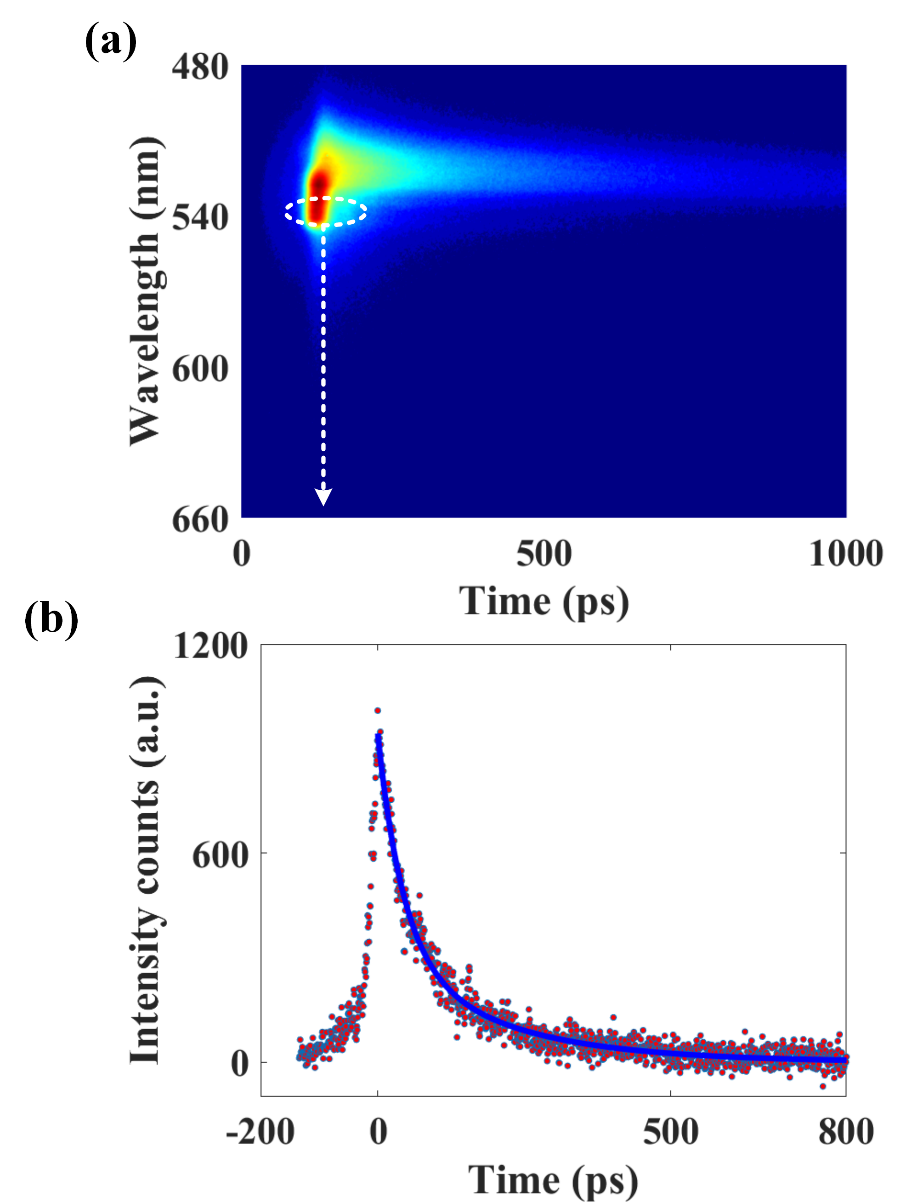


**Figure S4.** Lasing rate constant in core-only CdSe CQWs (donors). (a) The streak camera images when the fluence is beyond lasing threshold. (b) When lasing occurs, a fast recombination with the lifetime of ~16 ps is observed.

# Supplementary Note 1

- - 1. To evaluate the exciton flow efficiency based on the steady-state emission intensity of the donors, we introduce rate equations of the exciton dynamics. We assume that the recombination dynamics changes of donors in the CQW complex is solely attributed to the FRET process. We can build the rate equations^3,4^:
    2. $\begin{matrix} \frac{dN_{donor}^{pure}}{dt}=P_{donor}-\gamma_{donor}^{pure}N_{donor}^{pure} \\ \frac{dN_{donor}^{mix}}{dt}=P_{donor}-\gamma_{donor}^{mix}N_{donor}^{mix}=P_{donor}-(\gamma_{donor}^{pure}+\gamma_{FRET}^{donor})N_{donor}^{mix} \end{matrix}$ (S1)
    3. Where *P* is the generation rate for excited species in the donors and *N* is the population of excitons in the donors.
    4. The population of excitons in the donor can be derived from the steady−state solutions and expressed as:
    5. $N_{donor}^{pure}=\frac{P_{donor}}{\gamma_{donor}^{pure}};N_{donor}^{mix}=\frac{P_{donor}}{\gamma_{donor}^{pure}+\gamma_{FRET}^{donor}}$ (S2)
    6. The emission intensity is proportional to the radiative decay rate as follows:
    7. $I_{donor}^{pure}=N_{donor}^{pure}\gamma^{rad};I_{donor}^{mix}=N_{donor}^{mixed}\gamma^{rad}$ (S3)
    8. Where *γ^rad^* is the radiative recombination rate of excited species in the donors.
    9. Thus, we can calculate the outflowing efficiency of donors using the following:
    10. $\eta^{outflow}=1-N_{^{donor}}^{mixed}/N_{^{donor}}^{pure}=1-I_{donor}^{mixed}/I_{donor}^{pure}$ (S4)

# Supplementary Note 2

Here, we build up the FRET-coupled rate equation model to reproduce the exciton flow efficiency in the CQW complex. For simplification, a three-energy level system is adopted, and the stimulated emission process in the donors and the acceptors is modeled by introducing the ultrafast recombination rate, *k_lasing_*, when pumping fluence is higher than the corresponding threshold^5,6^. The set of rate equations is presented as follows.

$\begin{matrix} \bullet\bullet\bullet\bullet\bullet\bullet\bullet\bullet\bullet\bullet\bullet\bullet\bullet\bullet\bullet\bullet\bullet\bullet\bullet Donor\bullet\bullet\bullet\bullet\bullet\bullet\bullet\bullet\bullet\bullet\bullet\bullet\bullet\bullet\bullet\bullet\bullet\bullet\bullet\\ \frac{dN_{0}^{D}}{dt}= \left\{\begin{matrix} k_{ex}^{D}N_{1}^{D}-k_{p}^{D}(N_{0}^{D}-N_{2}^{D}) + k_{FRET}^{D\to A}N_{1}^{D}N_{0}^{A} & when N_{1}^{D} < N_{0}^{D} \\ k_{ex}^{D}N_{1}^{D}-k_{p}^{D}(N_{0}^{D}-N_{2}^{D}) + k_{la\mathrm{sing}}^{D}N_{1}^{D} + k_{FRET}^{D\to A}N_{1}^{D}N_{0}^{A} & when N_{1}^{D} > N_{0}^{D} \end{matrix}\right. \\ \frac{dN_{1}^{D}}{dt}= \left\{\begin{matrix} k_{rex}^{D}N_{2}^{D} - k_{ex}^{D}N_{1}^{D} - k_{FRET}^{D\to A}N_{1}^{D}N_{0}^{A} & when N_{1}^{D} < N_{0}^{D} \\ k_{rex}^{D}N_{2}^{D} - k_{ex}^{D}N_{1}^{D} - k_{la\mathrm{sing}}^{D}N_{1}^{D}- k_{FRET}^{D\to A}N_{1}^{D}N_{0}^{A} & when N_{1}^{D} > N_{0}^{D} \end{matrix}\right. \\ \frac{dN_{2}^{D}}{dt}= k_{p}^{D}(N_{0}^{D}-N_{2}^{D}) - k_{rex}^{D}N_{2}^{D} \\ \bullet\bullet\bullet\bullet\bullet\bullet\bullet\bullet\bullet\bullet\bullet\bullet\bullet\bullet\bullet\bullet\bullet Acceptor\bullet\bullet\bullet\bullet\bullet\bullet\bullet\bullet\bullet\bullet\bullet\bullet\bullet\bullet\bullet\bullet\bullet\bullet\bullet\\ \frac{dN_{0}^{A}}{dt}= \left\{\begin{matrix} k_{ex}^{A}N_{1}^{A}-k_{p}^{A}(N_{0}^{A}-N_{2}^{A}) - k_{FRET}^{D\to A}N_{1}^{D}N_{0}^{A} & when N_{1}^{D} < N_{0}^{D} \\ k_{ex}^{A}N_{1}^{A}-k_{p}^{A}(N_{0}^{A}-N_{2}^{A}) + k_{la\mathrm{sing}}^{A}N_{1}^{A} - k_{FRET}^{D\to A}N_{1}^{D}N_{0}^{A} & when N_{1}^{D} > N_{0}^{D} \end{matrix}\right. \\ \frac{dN_{1}^{A}}{dt}= \left\{\begin{matrix} k_{rex}^{A}N_{2}^{A} - k_{ex}^{A}N_{1}^{A} + k_{FRET}^{D\to A}N_{1}^{D}N_{0}^{A} & when N_{1}^{D} < N_{0}^{D} \\ k_{rex}^{A}N_{2}^{A} - k_{ex}^{A}N_{1}^{A} - k_{la\mathrm{sing}}^{A}N_{1}^{A}+ k_{FRET}^{D\to A}N_{1}^{D}N_{0}^{A} & when N_{1}^{D} > N_{0}^{D} \end{matrix}\right. \\ \frac{dN_{2}^{A}}{dt}= k_{p}^{A}(N_{0}^{A}-N_{2}^{A}) - k_{rex}^{A}N_{2}^{A} \end{matrix}$(S5)

Where, *k_p_* is the pumping rate; *N_0_* is the ground states; *N_1_* is the excitonic states; *N_2_* is the higher-energy state (i.e., multiple-exciton states) and will non-radiatively decay back to *N_1_* with a ultrafast rate of *k_rex_* (lifetime: 4 ps), *N_2_* will not contribute to the emission or FRET process in this model. *k_lasing_* is the rate of stimulated emission (lifetime: 16 ps).

After plug in the lifetime date from the time-resolved PL measurements (Figure S4), we can generate the dynamics of each states, and therefore, predict the FRET-mediated exciton flow. In the calculation, the initial input population (i.e., at time zero) for donors is [1, 0, 0], which corresponds to [*N_0_^D^*, *N_1_^D^*, *N_2_^D^*] and the initial input population (i.e., at time zero) for acceptors is [1, 0, 0], which corresponds to [*N_0_^A^*, *N_1_^A^*, *N_2_^A^*]. At any calculated data points, the convergence condition: *N_0_^D^*+*N_1_^D^*+*N_2_^D^*=1 *and N_0_^A^*+*N_1_^A^*+*N_2_^A^*=1 should be satisfied.

**References**

1. B. Guzelturk, *et al.* Nonradiative Energy Transfer in Colloidal CdSe Nanoplatelet Films. *Nanoscale* **7**, 2545 (2015).
2. J. Q. Grim, *et al.* Continuous-wave biexciton lasing at room temperature using solution-processed quantum wells. *Nature Nanotechnology* **9**, 891–895 (2014).
3. Daichi Kozawa, *et al.* Evidence for Fast Interlayer Energy Transfer in MoSe_2_/WS_2_ Heterostructures. *Nano Letters* **16**, 4087 (2016).
4. Colin M. Chow, *et al.* Unusual Exciton-Phonon Interactions at van der Waals Engineered Interfaces. *Nano Letters* **17**, 1194 (2017).
5. Kaifeng Wu, *et al.* Towards zero-threshold optical gain using charged semiconductor quantum dots. *Nature Nanotechnology* **12**, 1140 (2017).
6. Boris le Feber, *et al.* Colloidal-Quantum-Dot Ring Lasers with Active Color Control. *Nano Letters* **18**, 1028 (2018).
